# Choosing the target difference and undertaking and reporting the sample size calculation for a randomised controlled trial – the development of the DELTA^2^ guidance

**DOI:** 10.1186/s13063-018-2887-x

**Published:** 2018-10-10

**Authors:** William Sones, Steven A. Julious, Joanne C. Rothwell, Craig Robert Ramsay, Lisa V. Hampson, Richard Emsley, Stephen J. Walters, Catherine Hewitt, Martin Bland, Dean A. Fergusson, Jesse A. Berlin, Doug Altman, Luke David Vale, Jonathan Alistair Cook

**Affiliations:** 1 Centre for Statistics in Medicine, Nuffield Department of Orthopaedics, Rheumatology and Musculoskeletal Sciences, University of Oxford, Botnar Research Centre, Nuffield Orthopaedic Centre, Windmill Rd, Oxford, OX3 7LD UK; 2 Medical Statistics Group, ScHARR, The University of Sheffield, Regent Court, 30 Regent Street, Sheffield, S1 4DA UK; 3 Health Services Research Unit, University of Aberdeen, Health Sciences Building, Foresterhill, Aberdeen, AB25 2ZD UK; 4 Department of Mathematics and Statistics, Lancaster University, Lancaster, LA1 4YF UK; 5 Statistical Methodology and Consulting, Novartis Pharma AG, Basel, Switzerland; 6 Department of Biostatistics and Health Informatics, Institute of Psychiatry, Psychology and Neuroscience, King’s College London, De Crespigny Park, Denmark Hill, London, SE5 8AF UK; 7 Department of Health Sciences, Seebohm Rowntree Building, University of York, Heslington, York, YO10 5DD UK; 8 Clinical Epidemiology Program, Ottawa Hospital Research Institute, 501 Smyth Road, Box 201B, Ottawa, ON K1H 8L6 Canada; 9 Johnson & Johnson, One J&J Plaza, New Brunswick, NJ 08933 USA; 10 Health Economics Group, Institute of Health and Society, Newcastle University, Newcastle upon Tyne, UK

**Keywords:** Target difference, Clinically important difference, Sample size, Guidance, Randomised trial, Effect size, Delphi

## Abstract

**Background:**

A key step in the design of a randomised controlled trial is the estimation of the number of participants needed. The most common approach is to specify a target difference in the primary outcome between the randomised groups and then estimate the corresponding sample size. The sample size is chosen to provide reassurance that the trial will have high statistical power to detect the target difference at the planned statistical significance level. Alternative approaches are also available, though most still require specification of a target difference.

The sample size has many implications for the conduct of the study, as well as incurring scientific and ethical aspects. Despite the critical role of the target difference for the primary outcome in the design of a randomised controlled trial (RCT), the manner in which it is determined has received little attention. This article reports the development of the DELTA^2^ guidance on the specification and reporting of the target difference for the primary outcome in a sample size calculation for a RCT.

**Methods:**

The DELTA^2^ (Difference ELicitation in TriAls) project has five components comprising systematic literature reviews of recent methodological developments (stage 1) and existing funder guidance (stage 2), a Delphi study (stage 3), a 2-day consensus meeting bringing together researchers, funders and patient representatives (stage 4), and the preparation and dissemination of a guidance document (stage 5).

**Results:**

The project started in April 2016. The literature search identified 28 articles of methodological developments relevant to a method for specifying a target difference. A Delphi study involving 69 participants, along with a 2-day consensus meeting were conducted. In addition, further engagement sessions were held at two international conferences. The main guidance text was finalised on April 18, 2018, after revision informed by feedback gathered from stages 2 and 3 and from funder representatives.

**Discussion:**

The DELTA^2^ Delphi study identified a number of areas (such as practical recommendations and examples, greater coverage of different trial designs and statistical approaches) of particular interest amongst stakeholders which new guidance was desired to meet. New relevant references were identified by the review. Such findings influenced the scope, drafting and revision of the guidance. While not all suggestions could be accommodated, it is hoped that the process has led to a more useful and practical document.

**Electronic supplementary material:**

The online version of this article (10.1186/s13063-018-2887-x) contains supplementary material, which is available to authorized users.

## Background

Deciding upon an appropriate sample size is a key part of designing a randomised controlled trial (RCT) [1]. A sample size calculation is typically carried out. Too small a sample size could result in a difference being overlooked, whilst a sample size which is too large could be a waste of resources and also lead to spurious findings [2].

In the healthcare setting, the majority of RCTs adopt a conventional approach (Neyman–Pearson) to determining the sample size. With this approach, the sample size required for a RCT depends upon the magnitude of difference to be detected (the ‘target difference’) along with the risk of reporting a difference when none exists (type I error) and the risk of reporting no difference when a difference of the specified magnitude does exist (type II error). The sample size is highly dependent upon the magnitude of difference, the target difference or effect size, as it has often somewhat imprecisely been referred to. For example, reducing the target difference by a half, quadruples the sample size in a two-arm parallel-group trial with 1:1 allocation and a continuous outcome, which is assumed to be normally distributed [3].

Until recently [2], little has been published looking at methods to inform the choice of target difference. Initial guidance was prepared for standard (non-adaptive superiority two-arm parallel group) trials to be designed and analysed according to the Neyman–Pearson approach [4]. However, that guidance does not cover trials of different hypotheses (i.e. equivalence/non-inferiority trials), any complex designs (e.g. multi-arm or adaptive trials), or other alternative statistical approaches (such as Bayesian and precision based). It is clear that limitations in the scope and conception (as it was developed primarily for researchers) of the initial DELTA guidance means that it does not fully meet the needs of funders and researchers. The DELTA^2^ project [5] sought to address this gap. This paper reports on the development of the DELTA^2^ guidance.

## Aim and objectives

The DELTA^2^ project aimed to update and expand upon the previous DELTA guidance for researchers and funders to assist in the determination and corresponding reporting of the target difference (‘effect size’) when performing a sample size calculation for a RCT.

The specific project objectives were:

1. To review existing guidance provided by funders to researchers and scientific review panel/board members
2. To identify key methodological developments or changes in practice that have emerged since undertaking the comprehensive DELTA review [2, 6] and update the DELTA guidance
3. To determine the scope of guidance that would aid researchers and address funders’ needs
4. To achieve consensus on what structured guidance for choosing the target difference (effect size) should comprise
5. To identify future research needs

To achieve these objectives, a five-stage project was developed [5]. This publication briefly summarises the methods of the project, before proceeding to presenting the findings from stages 1–4, which informed the development of the guidance.

## Methods

A summary of the methods used in each stage are given below. The final guidance is available [7] and has been summarised in a companion paper (Cook JA, et al.: DELTA^2^ guidance on choosing the target difference and undertaking and reporting the sample size calculation for a randomised controlled trial - new guidance, forthcoming). The process of developing the guidance is described below.

### Stage 1 and 2. Identifying relevant literature and eliciting expert opinion

#### Literature search

A systematic review was performed to identify recent publications detailing novel approaches to determining the target difference for a RCT. Publications were identified using a systematic search within the PubMed database for articles published after the DELTA review (January 1, 2011) and March 31, 2016 [2, 6]. The search was restricted to journals where previous relevant methodological work in this area had been published [2, 6], supplemented by other leading journals in epidemiology, health economics, health research methodology, statistics and trials. Full details of the search strategy used can be found in Additional file 1.

In addition to the systematic review of publications, a review of existing online guidance provided by funding schemes and advisory bodies was performed.

#### Search for guidance

Guidance documents prepared by trial funding and advisory bodies to assist applicants applying for funding for a RCT were inspected for relevant text. Searches were carried out for documents associated with UK trial funding schemes run by the National Institute for Health Research (NIHR), including Efficacy and Mechanism Evaluation (EME), Health Technology Assessment (HTA), the Research for Patient Benefit (RfPB) Programme, Programme Grants for Applied Research (PGfAR), Public Health Research (PHR), Invention for Innovation (i4i), and Health Services and Delivery Research (HSDR), the Medical Research Council (MRC) Developmental Pathway Funding Scheme (DPFS), the Arthritis Research UK, the British Heart Foundation (BHF), Cancer Research UK (CRUK) (phase III clinical trial, new agent, population research), and the Wellcome Trust (Health Challenge Innovation Fund). The UK Health Research Authority's (HRA) documentation was searched. A search of guidance documents provided by the NIHR Research Design Service (RDS) was also performed. Similar searches were performed for leading international funding streams and regulatory agencies (Agency for Healthcare Research and Quality (AHRQ), Canadian Institutes of Health Research (CIHR), European Commission Horizon 2020 (H2020), Food and Drug Administration (FDA), Health Canada, National Health and Medical Research Council (NHMRC), National Institutes of Health (NIH), and Patient-Centered Outcomes Research Institute (PCORI)). Information contained within guidance for applicants applying for trial funding from funders and research advisory bodies regarding the choice of target difference was extracted.

#### Inclusion and exclusion criteria

The title and abstract of articles identified within the PubMed database search were independently assessed by two reviewers to identify publications worthy of further assessment. The full-text of a publication deemed worthy of further assessment was then analysed by a reviewer and included if considered to report a development not already encompassed within the previous DELTA review [2, 6].

#### Data extraction

Publications viewed to be of relevance were reviewed by an expert reviewer and aspects of interest noted. Information on undertaking a sample size calculation and the target difference choice was identified within the websites of trial funding and advisory bodies and the content assessed by two reviewers. A third (content expert) member of the team acted as arbiter for all disagreements or where further content expertise was required.

### Stage 3. Delphi study

A multi-round Delphi study was conducted with stakeholders known to have an interest in the design of RCTs. Participants were asked about what guidance was needed on specifying the target difference in a RCT sample size calculation. A 2-day consensus meeting and a one-off stakeholder engagement session were embedded within the Delphi study (stage 4; see below for details). Findings from the first Delphi round were considered by the 2-day consensus meeting to aid construction of a draft DELTA^2^ guidance document. A second-round questionnaire was sent with a hyperlink to the draft guidance document. Views and comments on the draft guidance overall, main body of the document, case studies, appendices and references were requested. Round 1 and 2 questionnaires are available in Additional file 2.

A group of known methods experts, the inclusion of which was informed by the DELTA review and findings from Stage 1, alongside representatives of key trial groups were invited to participate in the Delphi study. Representatives for groups including the UKCRC network of clinical trial units (CTUs), the MRC Hubs for Trials Methodology Research (HTMRs), NIHR/MRC/CRUK funding programme panels, the NIHR statistics group and the NIHR RDS were contacted using publicly available contact information and invited to participate. Participants comprised of one named individual per group (unit, board, MRC HTMR, RDS centre or programme; e.g. the director, chair or senior methodologist). These groups represent UK centres and networks of excellence that undertake high-quality trials research. As of July 1, 2016, there were 48 (fully or provisionally) registered CTUs, 5 MRC HTMRs and the 10 regions in the NIHR RDS in England, and the Research Design and Conduct Service in Wales.

Based upon the premise that a minimum of 30 participants would be required to participate in the Delphi process, and assuming one-third of invitees would agree to participate, it was felt that at least 90 invitations needed to be made. Due to the arbitrary nature of this target, no strict maximum was applied and 162 invitations were made.

### Stage 4. Two-day consensus meeting and one-off stakeholder engagement sessions

#### Two-day consensus meeting

Proposals as to the structure and content of the guidance, put forward as part of the first round Delphi process, in addition to literature developments and current guidance practices were presented to 25 stakeholders in a face-to-face, 2-day meeting. Additionally, a number of participants gave presentations that provided an overview of the use of specific approaches and/or personal experience of working in this area. Stakeholders, selected to cover a range of perspectives, areas of expertise and roles within RCT design, discussed and refined the proposal for the guidance document and reached a consensus on the format of the draft guidance document.

#### One-off stakeholder engagement sessions

To gain a broader range of opinions, engagement sessions were held at the Society for Clinical Trials (SCT) 37th Annual Meeting on May 17, 2016, Statisticians in the Pharmaceutical Industry (PSI) Conference on May 16, 2017, and on the Joint Statistical Meetings (JSM) Conference on August 1, 2017. Participants were invited to provide views on the scope and structure of the guidance needed and to offer constructive feedback on the draft guidance.

### Stage 5. Publication of guidance documentation

The provisional guidance was drafted upon completion of stages 1–4 and circulated amongst the DELTA^2^ members and Delphi participants for comments. UK Funder representatives will be asked to assess the guidance to ensure the document meets funding panel requirements and allow implementation of changes required for specific forms of publication.

## Results

### Stage 1. Systematic literature search results

**Fig. 1:**
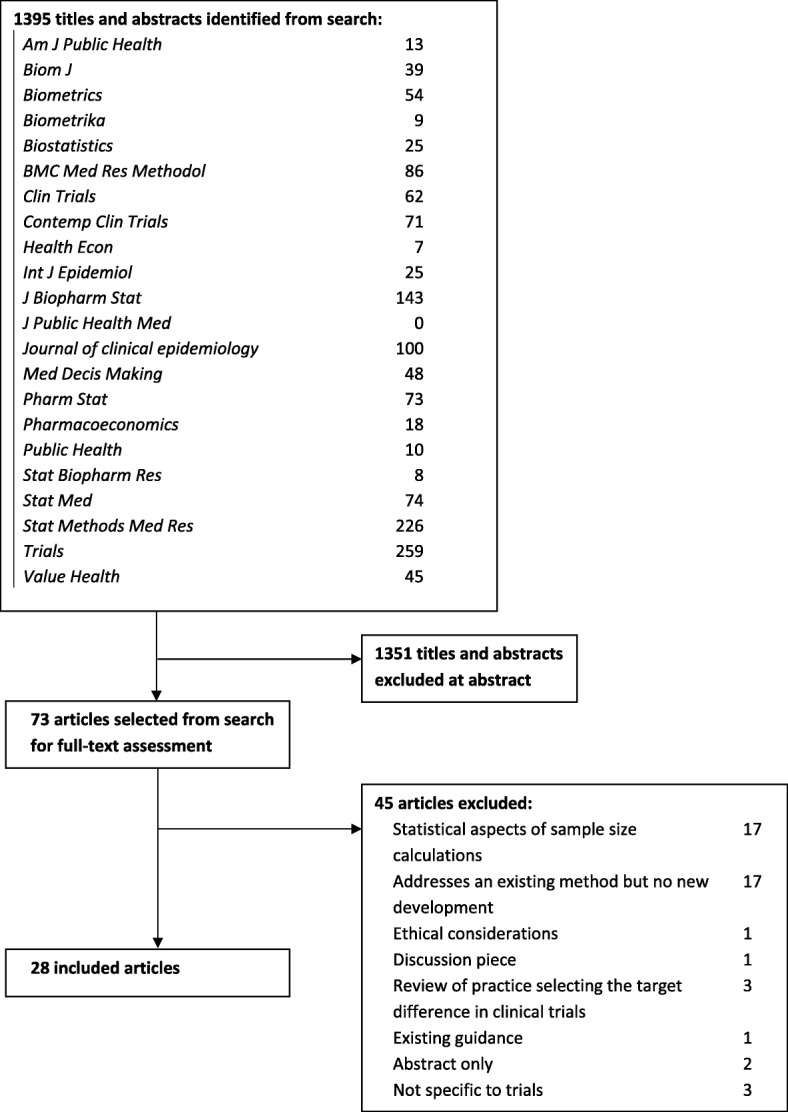
Flow diagram

The search identified 1395 potentially relevant reports (Fig. 1). Following the screening of titles and abstracts, 73 publications were full text assessed. Of these, 28 were included in the review as representing a development of one of the previously identified seven broad method types (Table 1 and Additional file 3). Minor developments were identified for the health economic (including cost-utility and value of information), opinion-seeking, pilot/preliminary study and standardised effect size approaches. No new methods were identified. Most developments (*n* = 17 articles) related to the use of variants of the value of information approach.

**Table 1:** Included studies from literature review of methodological development in methods for specifying a target difference

Study		Journal	Method	Methodological development
Hedayat	2015	*Biometrics*	Anchor	Variation in threshold-based approach to estimating the MID
Rouquette	2014	*Journal of Clinical Epidemiology*	Anchor	Use of item response model approach to calculate M(C)ID estimate from anchor assessment
Zhang	2015	*Journal of Clinical Epidemiology*	Anchor	Assessment of expressing MID as absolute and relative difference
Hollingworth	2013	*Clinical Trials*	HE (cost utility)	Assessment of cost-utility-based sample size approaches
Chen MH	2013	*Clinical Trials*	HE (VOI)	VOI for multistage adaptive trials from industry perspective
Andronis	2016	*Medical Decision making*	HE (VOI)	Expected value of sample information variant estimator
Breeze	2015	*Health economics*	HE (VOI)	ENBS from pharmaceutical perspective using value-based pricing
Hall	2012	*Medical Decision making*	HE (VOI)	Expected net present value of sample information approach
Jalal	2015	*Medical Decision making*	HE (VOI)	Meta modelling approach to calculating the expected value of sample information
Madan	2014	*Medical Decision making*	HE (VOI)	Efficient approach to calculating the expected value of partial perfect information (applicable to calculating the expected value of sample information)
Maroufy	2014	*Journal of Biopharmaceutical Statistics*	HE (VOI)	Method for calculating expected net gain of sampling
Mckenna	2011	*Medical Decision making*	HE (VOI)	Exploration of the role of value of sample information analysis
Menzies	2016	*Medical Decision making*	HE (VOI)	Expected value of sampling information estimator
Sadatsafavi	2013	*Health economics*	HE (VOI)	Expected value of sample information variant estimator
Streuten*	2013	*Pharmacoeconomics*	HE (VOI)	Comprehensive review of VOI methodological developments
Strong	2014	*Medical Decision making*	HE (VOI)	Meta modelling approach to calculating the expected value of sample information
Welton	2014	*Medical Decision making*	HE (VOI)	Application of expected value of sampling information to a cluster trial
Welton	2015	*Medical Decision making*	HE (VOI)	ENBS accounting for heterogeneity in treatment effects
Willan	2011	*Pharmacoeconomics*	HE (VOI)	Framework for exploring the perspective of societal decision-maker and industry
Willan	2012	*Health economics*	HE (VOI)	Accounting for between-study variation in value of information approach
Kirkby	2011	*BMC Medical Research Methodology*	Opinion seeking	Survey approach to estimate MCID in trial setting
Ross	2012	*BMC Medical Research Methodology*	Opinion seeking	Survey approach to estimate MCID with three treatment options
Chen H	2013	*Clinical Trials*	Pilot/Preliminary study	Comparison of approaches to using SD from preliminary study (e.g. pilot or phase 2 study)
Fay	2013	*Clinical Trials*	Pilot/Preliminary study	Variation in approach to using SD from preliminary study (e.g. pilot or phase 2 study)
Kirby	2015	*Pharmaceutical statistics*	Pilot/Preliminary study	Variation in approaches to discounting evidence from preliminary study
Sim	2012	*Journal of Clinical Epidemiology*	Pilot/Preliminary study	Inflation factor for pilot study SD estimate for use in trial sample size calculation
Whitehead	2016	*Statistics Method in Medical Research*	Pilot/Preliminary study	Assessment of size of pilot study needed to inform main trial
Valentine	2016	*JCE*	SES	Alternative effect size metric proposed

*This review summarised a substantial number of variants/generalisations in the VOI methods applicable to the sample size calculation of a RCT

*ENBS* expected value of net benefit sampling, *HE* health economics, *MID* minimally important difference *MCID* minimal clinically important difference, *SD* standard deviation, *SES* standardised effect size, *VOI* value of information

A number of helpful review articles that summarise different methods and variations in application were identified; these covered willingness to pay [8, 9] and value of information [10, 11] health economic based approaches, and estimation of the smallest worthwhile difference formulation of a minimal clinically important difference, which covered anchor, distribution, opinion-seeking and standardised effect size methods [12]. Identified articles on relevant topics (e.g. that address statistical aspects of sample size calculations or an existing method but contain no new development) were considered as potential references in the guidance document irrespective of whether they were included in this review.

### Stage 2. Search for existing guidance

A search for guidance documentation on the websites for the 15 trial funding and advisory bodies listed within the Methodology section was performed (Additional file 4). On the majority of websites, trial design guidance emphasised the need for applicants to provide sufficient detail to justify the chosen sample size, often going on to discuss techniques employed to calculate sample size but without providing any details or guidance on how this should be done. In particular, there was little specific guidance provided to assist researchers in specifying the target difference. The use of pilot/preliminary studies and ‘interim data’ was noted with limited further comment.

### Stage 3. Delphi study

Invitations to participate in the Delphi study were sent (by email on July 29, 2016) to 58 methods experts along with 104 named representatives of key trial groups (including UKCRC network CTUs, the MRC HTMRs, NIHR/MRC/CRUK funding programme panels, the NIHR statistics group and the NIHR RDS). Of the 162 individuals invited to participate, responses were received from 84 (52%), of whom 78 (48%) accepted the invitation and 6 formally declined to participate. Acceptance of the invitation was allowed up to October 10, 2016 (the last acceptance was received on October 4, 2016).

**Fig. 2:**
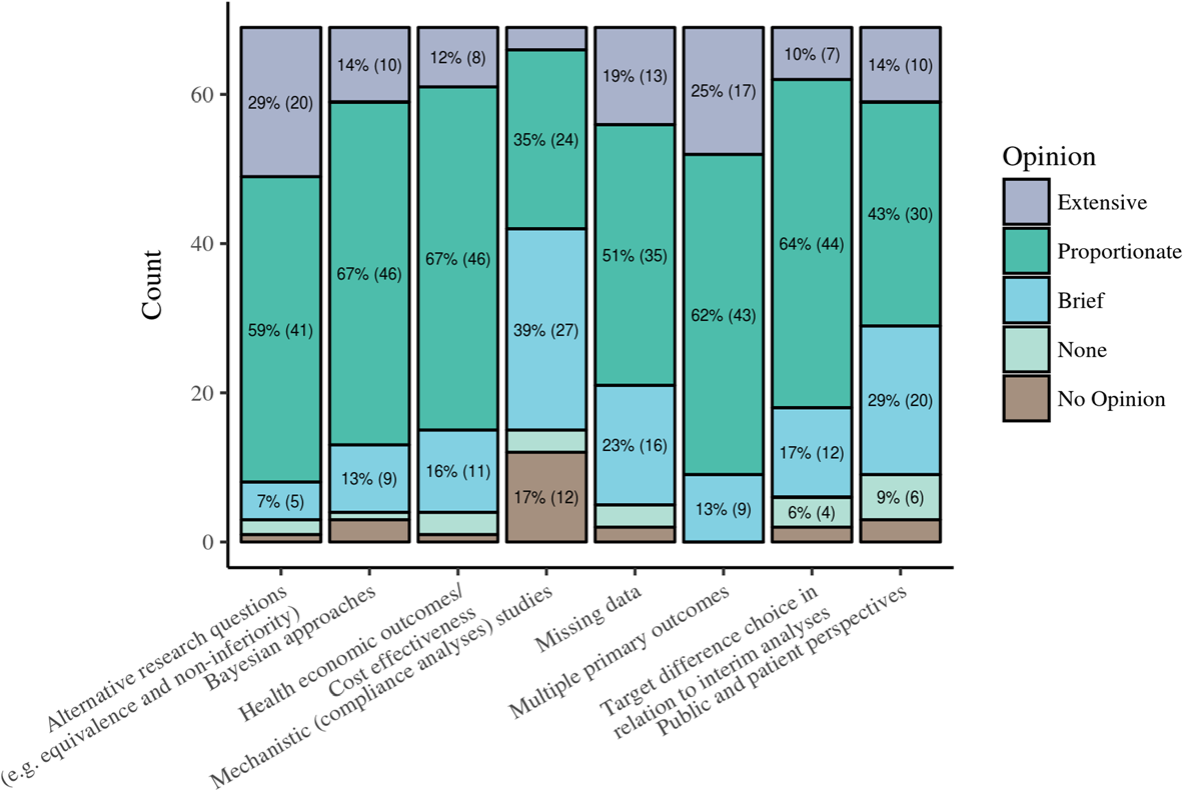
Round 1 Delphi online questionnaire responses. Specific topics to address within target difference estimation guidance

**Fig. 3:**
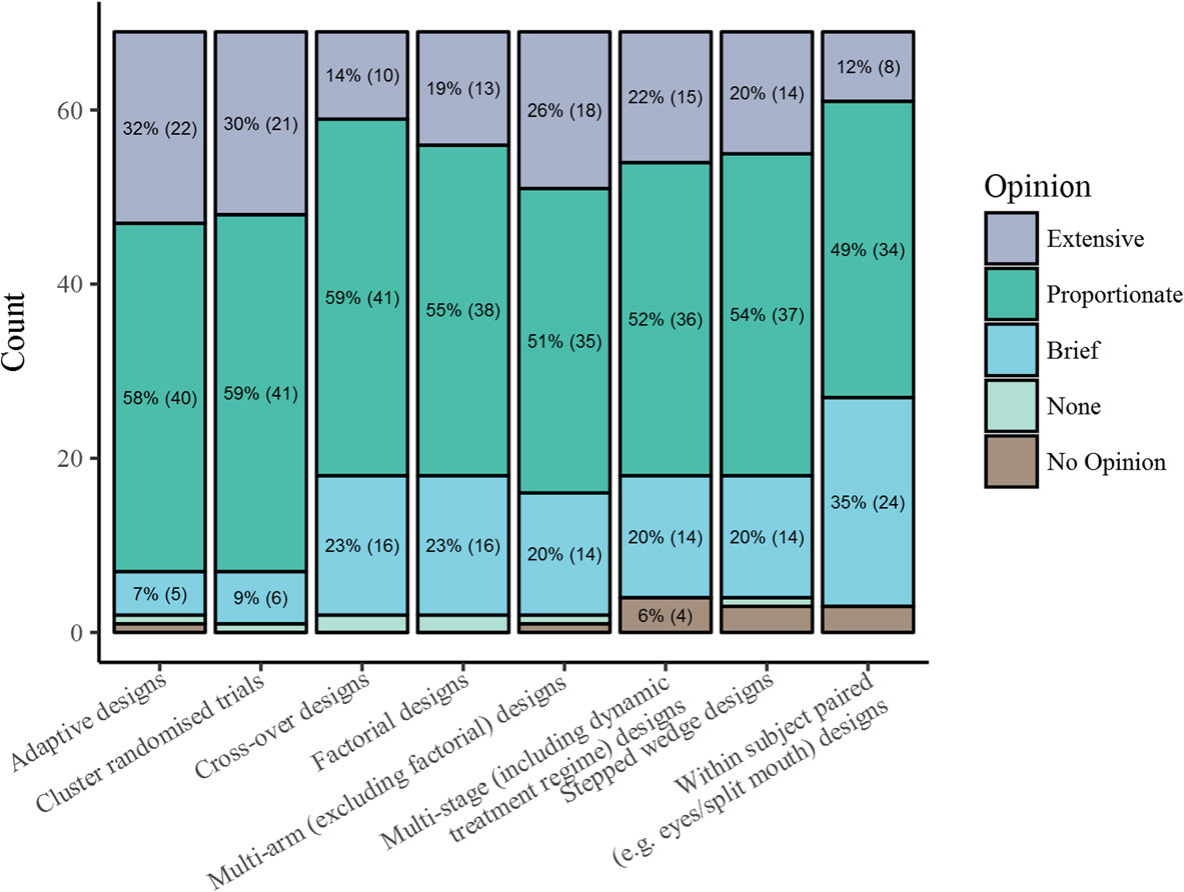
Round 1 Delphi online questionnaire responses. Alternative trial designs to address within target difference estimation guidance

The round 1 questionnaire was open for completion between August 11 and October 10, 2016. Of the 78 experts and representatives who agreed to participate, 69 (88%) completed the Round 1 questionnaire once invited by email whilst 9 did not complete it. The demographics of those who ultimately participated in the Delphi study are given in Table 2. Participants represented a range of RCT roles, with design, analysis and evaluating funding proposals well represented. The majority of participants (57 of the 69 who completed Round 1; 83%) were primarily affiliated with an academic institution, and the majority of participants were from the UK (55 of the 69 who completed Round 1; 80%). Views on whether specific topics and alternative designs (i.e. not a ‘standard’ two-arm, parallel-group design) should be covered within the guidance are given in Figs. 2 and 3. Delphi participants showed strongest support (≥ 25%) for extensive coverage on alternative research questions and handling multiple primary outcomes. Across most topics there was 50–70% support for proportionate coverage except for mechanistic studies and public and patient perspectives on the choice of the target difference. Regarding alternative study designs, the strongest support for extensive coverage was for adaptive designs, cluster randomised trials and multi-arm trials (all > 25%). Across all designs there was 50–60% support for proportionate coverage.

**Table 2:** Delphi participants’ demographics

Question	Response	Count (percentage of participants)
Your role in RCTs (select all that apply):	Involved in analysis of RCTs	42 (61%)
	Involved in RCT design (Collaborating Clinician)	7 (10%)
	Involved in RCT design (Lead/Chief Investigator)	23 (33%)
	Involved in RCT design (Statistician/Methodologist)	49 (71%)
	Other (Please specify)	16 (23%)
	Serves on a funding panel/board which evaluates applications for RCT funding	43 (62%)
Primary RCT related affiliation:	Academic institution	57 (83%)
	Contract research organisation	2 (3%)
	Funder of RCTs (e.g. NIHR in the UK or NIH in the US)	5 (7%)
	Healthcare provider (e.g. NHS in the UK)	3 (4%)
	Pharmaceutical/medical device company	2 (3%)
Where do you work? If you work across Europe or Internationally please choose the category in which the majority of your work is performed	Canada	3 (4%)
	Ireland	1 (1%)
	Other European Country	1 (1%)
	UK	55 (80%)
	US	9 (13%)
	Australasia	0 (0%)
	Other	0 (0%)

*NHS* National Health Service, *NIH* National Institutes of Health, *NIHR* National Institute for Health Research, *RCT* randomised controlled study

A total of 56 free-text comments were made covering personal views on specific topics, views on framing of research questions, and the audience that should be targeted for the guidance. Comments also included suggestions for additional trial designs to cover, references and case study topics.

**Fig. 4:**
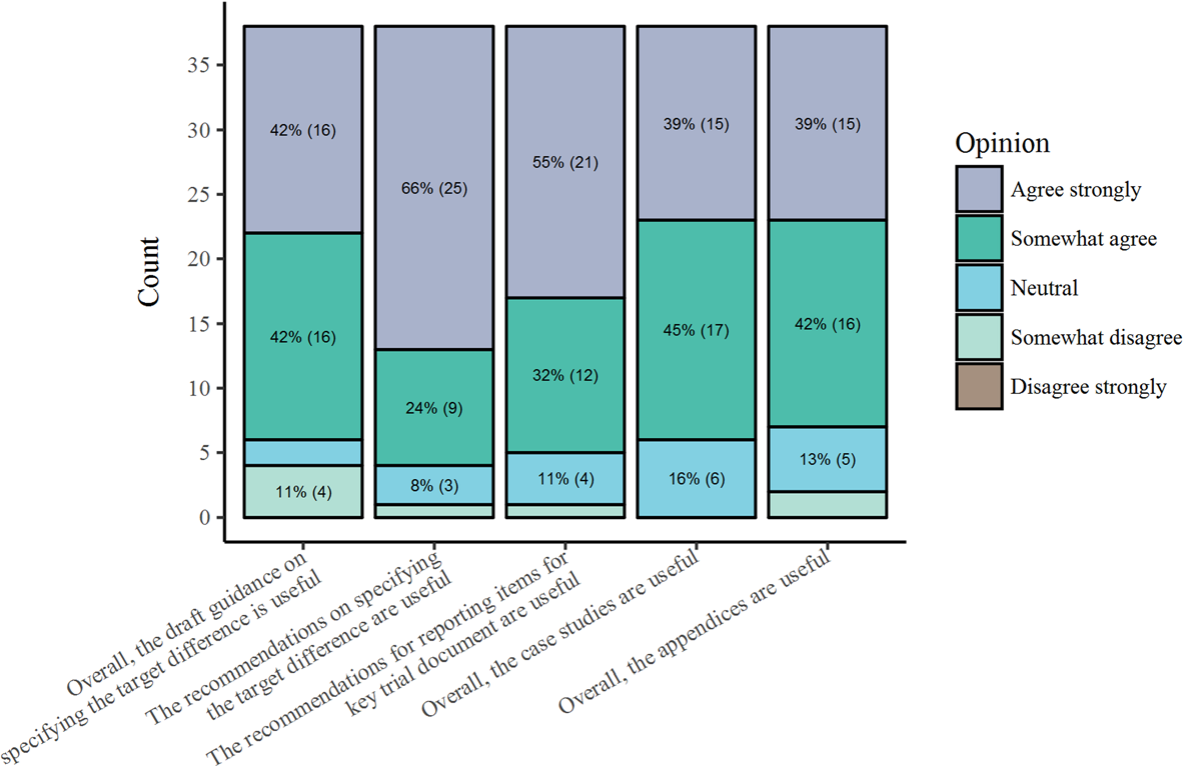
Round 2 Delphi online questionnaire responses

The Round 2 questionnaire was open for completion between September 1 and November 12, 2017. Only participants who completed Round 1 were invited to participate in Round 2 in which assessment of draft guidelines was required. Only two rounds were performed to fit with the project timescale and progress. Of the 69 participants invited to participate in Round 2, 38 (55%) completed Round 2. Findings from the Round 2 questionnaire are summarised in Fig. 4. Over 80% either ‘somewhat’ or ‘strongly’ agreed that the guidance was useful overall for the recommendations, case studies and appendices; 21 suggestions for improving the main text were made, 11 regarding the case studies and 9 on the appendices. In Round 2, 62 free-text comments were provided, which again covered a range of suggestions for improving the main text, adding an executive summary, improving the signposting of sections, views on the case studies and appendices, additional references, raising the issue of estimands, and personal views on various topics. Comments made in Round 1 and 2 questionnaires along with feedback from Stage 4 led to a substantial number of changes to the document prior to its finalisation. The most substantive being incorporating an executive summary and increasing the number of case studies.

### Stage 4. Two-day meeting and stakeholder engagement

An engagement session was held at SCT in May 2016, where the project was introduced, and views on the scope and broad content of the guidance were invited through audience participation. Following this, a 2-day workshop was held in Oxford on September 27–28, 2016, and involved 25 participants including CTU directors, study investigators, project funder representatives, funding panel members, researchers with experts in sample size methods, senior trial statisticians, and Public and Patient Involvement (PPI) representatives. The workshop included presentations of the findings from the initial two stages of the project, the SCT engagement session and Round 1 of the Delphi study, and focused upon decisions relating to the scope and content of the guidance. An initial structure for the first draft of the guidance was developed in light of the findings from the Round 1 questionnaire available at the time of the meeting. A revised structure was agreed by participants at the workshop. Drafting of individual sections was allocated to individuals. The recommendations on conducting a sample size calculation were initially drafted by JC. The various sections were then developed into the first full draft of the guidance by JC; this was circulated to all of the DELTA^2^ project group for comment, with the draft revised in light of these. An iterative process of comments and revisions was followed until the final version was agreed.

Subsequently, two further engagement sessions were held at PSI and JSM conferences. At the time of the session, the most current draft of the guidance was made available to participants. Both within and post-meeting feedback highlighted the need to consider the role of estimands and the minimum (statistically) detectable difference in the sample size calculation, leading to revisions in the guidance document. There was broad consensus, though not universal agreement, on the need for such guidance and the main topics it needed to cover from stakeholders across the various meetings and from the Delphi study. Differences of opinion tended to be about which topic needed to be covered and how important it was that they were covered.

### Stage 5. Finalisation, adaptation and dissemination

The draft guidance was reviewed by the representatives of the project’s funders (MRC-NIHR Methodology Advisory Group) on October 2, 2017. A number of revisions were made in light of feedback received from the advisory group and further feedback from the authors. The revised text of the main guidance was finalised on February 28, 2018. It was endorsed by the MRC-NIHR Methodology Advisory Group on March 12, 2018, with minor updating of references and the final version produced on April 18, 2017. Engagement with individual funders and funding programmes for the best way to utilise the guidance document and adapt to their needs is ongoing.

## Discussion

### Overview

The target difference is arguably the key value in a conventional sample size calculation but also the most difficult to choose. The DELTA^2^ project sought to produce more detailed guidance for researchers and funder representatives to aid researchers in making this choice and funder representatives in assessing the choice made. Building upon the DELTA guidance, a number of aspects were explored through engagement with stakeholders and the findings are summarised in this paper.

### Decisions on scope and content

As part of the process, we explored uncertainty about what methods for sample size determination should be covered. In particular, the views on two methods (value of information and standardised effect size-based approaches), which were included in the DELTA guidance, were debated and the inclusion reconsidered. There was general agreement that they should be included again but in particular the distinctive nature of the value of information approach required greater prominence. The need for some consideration of alternative statistical approaches (aside from the specification of the target difference per se) was also relatively strong. This resulted in specific appendices and boxes within the main guidance text covering more common alternative statistical methods and trial designs, and dealing with related aspects such as compliance analyses and missing data.

The need for more practical guidance was raised multiple times in various responses and in the engagement sessions. This led to two main additions in the final guidance document. First, 10 recommendations were made for specifying the target difference and a list of corresponding reporting items was included for when the conventional sample size approach is used. It is hoped that this will go some way to support researchers and funders undertaking and assessing sample size calculations. It is recognised that future adaptation to accommodate other study designs and statistical approaches will be needed. Second, a number of case studies were included, reflecting different trial designs and covering different conditions. Additional case studies could be added over time to provide a more complete coverage of the range of trial designs, statistical approaches and methods for specifying the target difference. Overall, the DELTA^2^ guidance is more comprehensive than the original DELTA guidance (and also more detailed, with over 25,000 compared to around 4000 words). It covers a much broader range of trials and approaches, with more practical guidance about how to undertake a sample size calculation for a RCT. A number of areas for further research were identified. Addressing these evidence gaps would help inform guidance for less common statistical approaches and trial designs.

### Strength and limitations

The main strength of this guidance lies in the extensive preparatory work undertaken in both the DELTA^2^ project and also the original DELTA work. The multiple avenues for engagement with stakeholders represent another strength, since this provided opportunities to solicit views on relevant topics and feedback on the draft guidance to be expressed by various stakeholders. A variety of methods were used to inform the development of the guidance document, including systematic reviews of the literature, a Delphi study using online questionnaires, engagement sessions with stakeholder groups and a 2-day workshop.

Participants in the various stages of the project were self-selected and may not be fully representative of all stakeholders. In particular, despite several attempts, there was limited involvement of industry statisticians with the exception of the PSI stakeholder meeting, and participants were mostly academic statisticians. Overall, those involved were possibly more methodologically interested than those who did not engage.

Timings of key meetings meant that flexibility was needed in the conduct of the stages and they were not carried out in a sequential manner as originally envisioned. The Delphi study only had 69 participants and had only two rounds, with a substantial drop off between rounds 1 and 2. Unlike other implementations of a Delphi study, a scoring system was not used to rank topics [13], nor was a formalised definition of consensus [14] used, as reflected in the more informal determination of consensus in this application.

The scope of some of the stages was purposely limited due to time and resource constraints. The journals searched for methodological developments were those thought to be most likely to publish new developments. It is possible that other developments have been published in other journals, which would have potentially been missed. Consulted stakeholders were predominantly based in the UK and the engagement sessions were limited in number and dependent upon acceptance of the proposal at the respective stakeholder meetings.

## Conclusions

The DELTA^2^ project identified a number of areas (such as practical recommendations and examples, greater coverage of alternative trial designs and statistical approaches) of particular interest amongst stakeholders, which the new guidance was designed to meet. Such findings influenced both the scope and drafting of the DELTA^2^ guidance document. Impact can be seen by the substantial changes in the document between the first and final versions. It is hoped that this process has led to a more useful and practical document.

## Additional files

Additional file 1: Search strategy details. (DOCX 13 kb)

Additional file 2: DELTA^2^ Delphi Questionnaires. (PDF 176 kb)

Additional file 3: List of included studies. (DOCX 43 kb)

Additional file 4: Findings from the review of relevant guidance. (DOCX 26 kb)

## Abbreviations

AHRQ: Agency for Healthcare Research and Quality
BHF: British Heart Foundation
CIHR: Canadian Institutes of Health Research
CRUK: Cancer Research UK
CTU: Clinical trial unit
DELTA: Difference ELicitation in TriAls
DPFS: Developmental Pathway Funding Scheme
EME: Efficacy and Mechanism Evaluation
ENBS: Expected value of net benefit sampling
FDA: Food and Drug Administration
HE: Health economics
HRA: Health Research Authority
HSDR: Health Services and Delivery Research
HTA: Health Technology Assessment
HTMR: Hubs for Trials Methodology Research
i4i: Invention for Innovation
ICH: International Council for Harmonisation of Technical Requirements for Pharmaceuticals for Human Use
IDREC: Inter-divisional Research Ethics Committee
JSM: Joint Statistical Meeting
MCID: Minimal clinically important difference
MID: Minimal important difference
MRC: Medical Research Council
MRP: Methodology Research Programme
NHMRC: National Health and Medical Research Council
NIH: National Institutes of Health
NIHR: National Institute for Health Research
PCORI: Patient-Centered Outcomes Research Institute
PFA: PCORI Funding Announcement
PGfAR: Programme Grants for Applied Research
PHR: Public Health Research
PPI: Patients and public involvement
PSI: Statisticians in the Pharmaceutical Industry
RCT: Randomised controlled trial
RDS: Research Design Service
RfPB: Research for Patient Benefit Programme
SCT: Society for Clinical Trials
SD: Standard deviation
UKCRC: UK Clinical Research Collaboration
VOI: Value of information

## References

[1] Bland JM (2009). The tyranny of power: is there a better way to calculate sample size?. BMJ.

[2] Cook JA (2014). Assessing methods to specify the target difference for a randomised controlled trial: DELTA (Difference ELicitation in TriAls) review. Health Technol Assess.

[3] Julious S (2010). Sample sizes for clinical trials.

[4] Cook J (2015). Specifying the target difference in the primary outcome for a randomised controlled trial: guidance for researchers. Trials.

[5] Cook JA (2017). Choosing the target difference (‘effect size’) for a randomised controlled trial - DELTA^2^ guidance protocol. Trials.

[6] Hislop J (2014). Methods for specifying the target difference in a randomised controlled trial: the Difference ELicitation in TriAls (DELTA) systematic review. PLoS Med.

[7] Cook, J.A., et al. Choosing the target difference and undertaking and reporting the sample size calculation for a randomised controlled trial - DELTA^2^ guidance for researchers and funder representatives. 2018 https://www.csm.ox.ac.uk/research/methodology-research/delta2/delta2-output. Accessed 20 Aug 2018.

[8] Glick HA (2011). Sample size and power for cost-effectiveness analysis (part 1). Pharmacoeconomics.

[9] Glick HA (2011). Sample size and power for cost-effectiveness analysis (part 2): the effect of maximum willingness to pay. Pharmacoeconomics.

[10] Willan AR (2011). Sample size determination for cost-effectiveness trials. Pharmacoeconomics.

[11] Wilson EC (2015). A practical guide to value of information analysis. Pharmacoeconomics.

[12] Ferreira ML (2012). A critical review of methods used to determine the smallest worthwhile effect of interventions for low back pain. J Clin Epidemiol.

[13] Pezold ML (2016). Defining a research agenda for patient-reported outcomes in surgery: using a Delphi survey of stakeholders. JAMA Surg.

[14] Williamson PR (2012). Developing core outcome sets for clinical trials: issues to consider. Trials.

