# Correction to: Choosing the target difference and undertaking and reporting the sample size calculation for a randomised controlled trial – the development of the DELTA2 guidance

**DOI:** 10.1186/s13063-019-3809-2

**Published:** 2019-10-24

**Authors:** William Sones, Steven A. Julious, Joanne C. Rothwell, Craig Robert Ramsay, Lisa V. Hampson, Richard Emsley, Stephen J. Walters, Catherine Hewitt, Martin Bland, Dean A. Fergusson, Jesse A. Berlin, Doug Altman, Luke David Vale, Jonathan Alistair Cook

**Affiliations:** 1 Centre for Statistics in Medicine, Nuffield Department of Orthopaedics, Rheumatology and Musculoskeletal Sciences, University of Oxford, Botnar Research Centre, Nuffield Orthopaedic Centre, Windmill Rd, Oxford, OX3 7LD UK; 2 Medical Statistics Group, ScHARR, The University of Sheffield, Regent Court, 30 Regent Street, Sheffield, S1 4DA UK; 3 Health Services Research Unit, University of Aberdeen, Health Sciences Building, Foresterhill, Aberdeen, AB25 2ZD UK; 4 Department of Mathematics and Statistics, Lancaster University, Lancaster, LA1 4YF UK; 5 Statistical Methodology and Consulting, Novartis Pharma AG, Basel, Switzerland; 6 Department of Biostatistics and Health Informatics, Institute of Psychiatry, Psychology and Neuroscience, King ’ s College London, De Crespigny Park, Denmark Hill, London, SE5 8AF UK; 7 Department of Health Sciences, Seebohm Rowntree Building, University of York, Heslington, York, YO10 5DD UK; 8 Clinical Epidemiology Program, Ottawa Hospital Research Institute, 501 Smyth Road, Box 201B, Ottawa, ON K1H 8L6 Canada; 9 Johnson & Johnson, One J&J Plaza, New Brunswick, NJ 08933 USA; 10 Health Economics Group, Institute of Health and Society, Newcastle University, Newcastle upon Tyne, UK

**Correction to: Trials (2018) 19:542**

**https://doi.org/10.1186/s13063-018-2887-x**

Following publication of the original article [1], we have been notified of a few mistakes:

- Figure 1:
  - “1351 titles and abstracts” should read “1322 titles and abstracts”
  - "Stat Biopharm Res" value should be "8" instead of "18"
  - "Stat Med" value should be "226" instead of "74"
  - "Stat Methods Med Res" value should be "74" instead of "226"
  - Results section, Stage 5, first paragraph, the phrase:
    - “It was endorsed by the MRC-NIHR Methodology Advisory Group on March 12, 2018, with minor updating of references and the final version produced on April 18, **2017**” should read
    - “It was endorsed by the MRC-NIHR Methodology Advisory Group on March 12, 2018, with minor updating of references and the final version produced on April 18, **2018**”.
