# Supplementary material for: Choosing the target difference and undertaking and reporting the sample size calculation for a randomised controlled trial – the development of the DELTA2 guidance
Source: Trials. 2018 Oct 10;19:542. doi: 10.1186/s13063-018-2887-x (PMC6180499; doi:10.1186/s13063-018-2887-x)
Supplement: Supplementary file 1 — Search strategy details. (DOCX 13 kb) [file 13063_2018_2887_MOESM1_ESM.docx]

**Additional file 1 Search strategy details**

Search terms used were sample size *or* target difference *or* effect size *or* important difference *or* detectable difference *or* power calculation *or* value of information *or* value of perfect information *or* value of partial perfect information *or* value of sampling information *or* expected net gain.

The period searched was 1^st^ January 2011 to 31^st^ March 2016.

A search was performed of the articles from the following journals for relevant publications:

American Journal of Public Health, Biometrical Journal, Biometrics, Biometrika, Biostatistics, BMC Medical Research Methodology, Clinical Trials, Contemporary Clinical Trials, Health Economics, International Journal of Epidemiology, Journal of Biopharmaceutical Statistics, Journal of Public Health Medicine, Journal of Clinical Epidemiology, Medical Decision Making, Pharmaceutical Statistics, PharmacoEconomics, Public Health, Statistics in Biopharmaceutical Research, Statistics in Medicine, Statistical Methods in Medical Research, Trials, and Value in Health.
