# Supplementary material for: Choosing the target difference and undertaking and reporting the sample size calculation for a randomised controlled trial – the development of the DELTA2 guidance
Source: Trials. 2018 Oct 10;19:542. doi: 10.1186/s13063-018-2887-x (PMC6180499; doi:10.1186/s13063-018-2887-x)
Supplement: Supplementary file 2 — DELTA2 Delphi Questionnaires. (PDF 176 kb) [file 13063_2018_2887_MOESM2_ESM.pdf]

DELTA2 Survey

Page 1: Introduction

Randomised controlled trials (RCT) are widely considered to be the optimal study design to assess comparative clinical efficacy and effectiveness, along with the cost implications of health interventions. Central to the validity of a RCT, is an *a-priori* sample size calculation which ensures the study has a reasonable chance to achieve its pre-specified objectives. Typically the sample size is calculated in order to ensure it is likely that a particular the magnitude of a difference between groups ("target difference" or "effect size") will be detectable.

Current published guidance on specifying the target difference is limited. See for example (Cook et al.) which covers only standard (superiority two-arm parallel) group trials and does not address Bayesian approaches or more complex trial designs (e.g. multi-arm trials).

The role of this survey is to determine the scope of guidance that researcher's and funders would find useful.

By completing this short survey (10 questions), which takes around 10 min to complete, you will help shape the guidance.

Thank you for your time and support, it's greatly appreciated!

I confirm I am over 18 years old and agree to take part in this study.

- ○ Yes
- ○ No

Page 2: About you

Your role in RCTs (select all that apply): ✱ *Required*

Please select at least 1 answer(s).

- ☐ Involved in RCT design (Lead/Chief Investigator)
- ☐ Involved in RCT design (Statistician/Methodologist)
- ☐ Involved in RCT design (Collaborating Clinician)
- ☐ Involved in analysis of RCTs
- ☐ Serves on a funding panel/board which evaluates applications for RCT funding
- ☐ Other (Please specify)

If you selected Other, please specify:

Primary RCT related affiliation: ✱ *Required*

- ○ Academic institution
- ○ Healthcare provider (e.g. NHS in the UK)
- ○ Funder of RCTs (e.g. NIHR in the UK or NIH in the US)
- ○ Pharmaceutical/medical device company
- ○ Contract research organsiation
- ○ Patient and public representative
- ○ Other (Please specify)

If you selected Other, please specify:

Where do you work? If you work across Europe or Internationally please choose the category in which the majority of your work is performed. ✱
Required

- ◯ UK
- ◯ Ireland
- ◯ Other European Country
- ◯ US
- ◯ Canada
- ◯ Australasia
- ◯ Other (Please specify)

If you selected Other, please specify:

Page 3: Scope of guidance

Types of studies

Guidance for specifying the target difference for a phase III/IV (often called "definitive" or "confirmatory") trial needs to be dealt with separately from early phase, pilot or feasibility trials.

	Degree of opinion ✱ *Required*					
	No opinion	Disagree strongly	Somewhat disagree	Neutral	Somewhat agree	Agree strongly
Choose one	☐	☐	☐	☐	☐	☐

Methods for specifying target difference

Should the following approaches be considered a formal method and covered within the guidance?

More info

	Degree of opinion ✱ *Required*					
	No opinion	Disagree strongly	Somewhat disagree	Neutral	Somewhat agree	Agree strongly
"Standardised effect size" (further information available above)	☐	☐	☐	☐	☐	☐
"Value of information" (further information available above)	☐	☐	☐	☐	☐	☐

Page 4: Scope of guidance

Special topics

Degree of coverage required for the following special topics in relation to specification of the target difference:

	Level of guidance required ✱ *Required*				
	No Opinion	None	Brief	Proportionate	Extensive
Alternative research questions (e.g. equivalence and non-inferiority)	○	○	○	○	○
Bayesian approaches	○	○	○	○	○
Health economic outcomes/Cost effectiveness	○	○	○	○	○
Mechanistic (compliance analyses) studies	○	○	○	○	○
Missing data	○	○	○	○	○
Multiple primary outcomes	○	○	○	○	○
Target difference choice in relation to interim analyses	○	○	○	○	○
Public and patient perspectives	○	○	○	○	○
Other (please detail below)	○	○	○	○	○

Other special topics with brief reason for inclusion.

Page 5: Scope of guidance

Complex designs

Degree of coverage for trials with more complex designs:

	Level of guidance required ✱ *Required*				
	No Opinion	None	Brief	Proportionate	Extensive
Adaptive designs					
Cluster randomised trials					
Cross-over designs					
Factorial designs					
Multi-arm (excluding factorial) designs					
Multi-stage (including dynamic treatment regime) designs					
Stepped wedge designs					
Within subject paired (e.g. eyes/split mouth) designs					
Other (please specify below)					

Other designs with reason for inclusion.

Page 6: Existing guidance

Please briefly review the existing guidance paper on this topic.

Specifying the target difference in the primary outcome for a randomised controlled trial: guidance for researchers (Cook et al. 2015).

The existing paper is useful: ✱ *Required*

Please don't select more than 1 answer(s) per row.

Please select at least 1 answer(s).

	Disagree strongly	Somewhat disagree	Neutral	Somewhat agree	Agree strongly
Choose one:	☐	☐	☐	☐	☐

How could it be improved?

Page 7: Additional comments

Are there any other comments related to guidance on specifying target difference for randomised trials which you would like to make?

Page 8: Thanks

Thanks for completing this survey which is very helpful and greatly appreciated!

DELTA2 Delphi survey Round 2

Page 1: Introduction

Previously, you kindly agreed to take part in a Delphi study as part of the DELTA^2 project.

This project seeks to produce consensus guidance on specifying the target difference in a randomised trial sample size calculation. Typically, the sample size is calculated in order to ensure it is likely that a particular magnitude of a difference between groups ("target difference" or "effect size") can be detectable. As you may recall DELTA^2 was a UK MRC/NIHR Methodology Research Programme Advisory Group funded work to produce guidance to enable further development upon previous guidance and literature on this topic, which although very useful is limited in scope (See for example (Cook et al. 2015)).

A summary of the Delphi round 1 findings, along with other projects findings including feedback from stakeholders engagement session are available here. Following your response to the first round of the Delphi, a two-day workshop was held in Oxford and initial guidance was drafted. Subsequently, drawing upon extensive feedback from group members and stakeholders (we held invited sessions at this year's PSI and JSM annual conferences), the draft guidance was revised extensively. To aid further refinement of the document before finalising, we would now be grateful for your feedback upon the current version of the guidance.

The role for you as a Delphi participant within this round 2 (and anticipated to the last) survey, is to ascertain your opinion upon the latest draft version of the guidance. We would like to get your views and suggestions on the key aspects of it (e.g. recommendations and case studies). A pdf

document of the latest version of the guidance is available here.

By completing this short survey (10 questions) you will help shape the final guidance.

Thank you for your time and support, it's greatly appreciated!

Page 2: Review of draft guidance, main body

How can the main body of the guidance document (pages 1-18) be improved?

Overall, the draft guidance on specifying the target difference is useful:

	Disagree strongly	Somewhat disagree	Neutral	Somewhat agree	Agree strongly
Choose one:	☐	☐	☐	☐	☐

The recommendations on specifying the target difference (Box 3, page 11) are useful:

	Disagree strongly	Somewhat disagree	Neutral	Somewhat agree	Agree strongly
Choose one:	☐	☐	☐	☐	☐

The recommendations for reporting items for key trial document are useful (Section 4/Figure 1, pages 16-18):

	Disagree strongly	Somewhat disagree	Neutral	Somewhat agree	Agree strongly
Choose one:	☐	☐	☐	☐	☐

How can the main body of the guidance document (pages 1-18) be improved?

Page 3: Review of draft guidance, case studies/appendices

Please briefly review the case studies/appendices sections of the draft guidance report.

Overall, the case studies (pages 19-31) are useful:

	Disagree strongly	Somewhat disagree	Neutral	Somewhat agree	Agree strongly
Choose one:	☐	☐	☐	☐	☐

How can the case studies be improved?

Overall, the appendices (pages 32-46) are useful:

	Disagree strongly	Somewhat disagree	Neutral	Somewhat agree	Agree strongly
Choose one:	☐	☐	☐	☐	☐

How can the appendices be improved?

Do you think there are any key relevant references missing from the document (pages 49-56)?

Page 4: Additional comments

Please use this box to provide any additional comments related to guidance on specifying target difference for randomised trials which you would like to make.

Page 5: Thanks

Thanks for completing this survey which is very helpful and greatly appreciated!

If you would like to further assist the DELTA^2 project by providing a case-study of a RCT sample size calculation, please contact jonathan.cook@ndorms.ox.ac.uk

---
