# Supplementary material for: Choosing the target difference and undertaking and reporting the sample size calculation for a randomised controlled trial – the development of the DELTA2 guidance
Source: Trials. 2018 Oct 10;19:542. doi: 10.1186/s13063-018-2887-x (PMC6180499; doi:10.1186/s13063-018-2887-x)
Supplement: Supplementary file 3 — List of included studies. (DOCX 43 kb) [file 13063_2018_2887_MOESM3_ESM.docx]

(1-28)

**List of included studies (n=28)**

1. Hedayat AS, Wang J, Xu T. Minimum clinically important difference in medical studies. Biometrics. 2015;71(1):33-41.

2. Rouquette A, Blanchin M, Sebille V, Guillemin F, Cote SM, Falissard B, et al. The minimal clinically important difference determined using item response theory models: an attempt to solve the issue of the association with baseline score. Journal of clinical epidemiology. 2014;67(4):433-40.

3. Zhang Y, Zhang S, Thabane L, Furukawa TA, Johnston BC, Guyatt GH. Although not consistently superior, the absolute approach to framing the minimally important difference has advantages over the relative approach. Journal of clinical epidemiology. 2015;68(8):888-94.

4. Hollingworth W, McKell-Redwood D, Hampson L, Metcalfe C. Cost-utility analysis conducted alongside randomized controlled trials: are economic end points considered in sample size calculations and does it matter? Clinical trials. 2013;10(1):43-53.

5. Chen H, Zhang N, Lu X, Chen S. Caution regarding the choice of standard deviations to guide sample size calculations in clinical trials. Clinical trials. 2013;10(4):522-9.

6. Chen MH, Willan AR. Determining optimal sample sizes for multistage adaptive randomized clinical trials from an industry perspective using value of information methods. Clinical trials. 2013;10(1):54-62.

7. Andronis L, Barton PM. Adjusting Estimates of the Expected Value of Information for Implementation: Theoretical Framework and Practical Application. Medical decision making : an international journal of the Society for Medical Decision Making. 2016;36(3):296-307.

8. Breeze P, Brennan A. Valuing Trial Designs from a Pharmaceutical Perspective Using Value-Based Pricing. Health economics. 2015;24(11):1468-82.

9. Hall PS, Edlin R, Kharroubi S, Gregory W, McCabe C. Expected net present value of sample information: from burden to investment. Medical decision making : an international journal of the Society for Medical Decision Making. 2012;32(3):E11-21.

10. Jalal H, Goldhaber-Fiebert JD, Kuntz KM. Computing Expected Value of Partial Sample Information from Probabilistic Sensitivity Analysis Using Linear Regression Metamodeling. Medical decision making : an international journal of the Society for Medical Decision Making. 2015;35(5):584-95.

11. Madan J, Ades AE, Price M, Maitland K, Jemutai J, Revill P, et al. Strategies for efficient computation of the expected value of partial perfect information. Medical decision making : an international journal of the Society for Medical Decision Making. 2014;34(3):327-42.

12. McKenna C, Claxton K. Addressing adoption and research design decisions simultaneously: the role of value of sample information analysis. Medical decision making : an international journal of the Society for Medical Decision Making. 2011;31(6):853-65.

13. Steuten L, van de Wetering G, Groothuis-Oudshoorn K, Retèl V. A Systematic and Critical Review of the Evolving Methods and Applications of Value of Information in Academia and Practice. PharmacoEconomics. 2013;31(1):25-48.

14. Strong M, Oakley JE, Brennan A. Estimating multiparameter partial expected value of perfect information from a probabilistic sensitivity analysis sample: a nonparametric regression approach. Medical decision making : an international journal of the Society for Medical Decision Making. 2014;34(3):311-26.

15. Maroufy V, Marriott P, Pezeshk H. An optimization approach to calculating sample sizes with binary responses. Journal of biopharmaceutical statistics. 2014;24(4):715-31.

16. Menzies NA. An Efficient Estimator for the Expected Value of Sample Information. Medical decision making : an international journal of the Society for Medical Decision Making. 2016;36(3):308-20.

17. Sadatsafavi M, Marra C, Bryan S. Two-level resampling as a novel method for the calculation of the expected value of sample information in economic trials. Health economics. 2013;22(7):877-82.

18. Welton NJ, Madan JJ, Caldwell DM, Peters TJ, Ades AE. Expected value of sample information for multi-arm cluster randomized trials with binary outcomes. Medical decision making : an international journal of the Society for Medical Decision Making. 2014;34(3):352-65.

19. Welton NJ, Soares MO, Palmer S, Ades AE, Harrison D, Shankar-Hari M, et al. Accounting for Heterogeneity in Relative Treatment Effects for Use in Cost-Effectiveness Models and Value-of-Information Analyses. Medical decision making : an international journal of the Society for Medical Decision Making. 2015;35(5):608-21.

20. Willan AR, Eckermann S. Accounting for between-study variation in incremental net benefit in value of information methodology. Health economics. 2012;21(10):1183-95.

21. Willan AR, Eckermann S. Value of information and pricing new healthcare interventions. Pharmacoeconomics. 2012;30(6):447-59.

22. Kirkby HM, Wilson S, Calvert M, Draper H. Using e-mail recruitment and an online questionnaire to establish effect size: A worked example. BMC medical research methodology. 2011;11:89.

23. Ross S, Milne J, Dwinnell S, Tang S, Wood S. Is it possible to estimate the minimal clinically important treatment effect needed to change practice in preterm birth prevention? Results of an obstetrician survey used to support the design of a trial. BMC medical research methodology. 2012;12:31.

24. Fay MP. An alternative property for evaluating sample size for normal data using preliminary data. Clinical trials. 2013;10(6):990-1.

25. Kirby S, Burke J, Chuang-Stein C, Sin C. Discounting phase 2 results when planning phase 3 clinical trials. Pharmaceutical statistics. 2012;11(5):373-85.

26. Whitehead AL, Julious SA, Cooper CL, Campbell MJ. Estimating the sample size for a pilot randomised trial to minimise the overall trial sample size for the external pilot and main trial for a continuous outcome variable. Statistical methods in medical research. 2016;25(3):1057-73.

27. Valentine JC, Aloe AM. How to communicate effect sizes for continuous outcomes: a review of existing options and introducing a new metric. Journal of clinical epidemiology. 2016;72:84-9.

28. Sim J, Lewis M. The size of a pilot study for a clinical trial should be calculated in relation to considerations of precision and efficiency. Journal of clinical epidemiology. 2012;65(3):301-8.
