# Supplementary material for: Choosing the target difference and undertaking and reporting the sample size calculation for a randomised controlled trial – the development of the DELTA2 guidance
Source: Trials. 2018 Oct 10;19:542. doi: 10.1186/s13063-018-2887-x (PMC6180499; doi:10.1186/s13063-018-2887-x)
Supplement: Supplementary file 4 — Findings from the review of relevant guidance. (DOCX 26 kb) [file 13063_2018_2887_MOESM4_ESM.docx]

**Additional file 4:** Review of relevant guidance available on funder websites

| **Arthritis Research UK** |  | No specific relevant guidance |
| --- | --- | --- |
| **BHF** | <https://www.bhf.org.uk/-/media/files/research/clinical-study-guidelines_interventional-study-(1).pdf?la=en> | Clinical study guidelines:  “Proposed sample size. Specify the number of participants and centres (including both control and treatment groups).” And  “Power calculations. Give details of the estimated effect size, power and/or precision employed in the calculation. Justify the estimated effect size and the assumptions underlying the sample size calculations.” |
| **HRA** | <http://www.hra.nhs.uk/documents/2014/05/guidance-questions-considerations-clinical-trials.pdf> | “If researchers are too optimistic about the size of the expected treatment difference,  the sample size will be too small, and the study may not have sufficient power to detect the minimum clinically important difference – in which case it will be inconclusive.” |
| **MRC** | <https://www.mrc.ac.uk/documents/pdf/complex-interventions-guidance/> | Generic:  “For small studies, which may not produce statistically significant results if the initial assumptions about effect sizes, recruitment rates, etc., were over-optimistic, pooling makes the results far more useful.”  Pilot/preliminary study:  “The feasibility and piloting stage includes testing procedures for their acceptability, estimating the likely rates of recruitment and retention of subjects, and the calculation of appropriate sample sizes. ….Pilot study results should be interpreted cautiously when making assumptions about the required sample size, likely response rates, etc., when the evaluation is scaled up. Effects may be smaller or more variable and response rates lower when the intervention is rolled out across a wider range of settings.” |
| **RDS** | <http://www.rds-london.nihr.ac.uk/RDSLondon/media/RDSContent/files/PDFs/Calculating-Sample-Size.pdf> | “Justifying the number of participants – or the “sample size” – is a vital part of planning a clinical trial for ethical reasons” and “In order to work out the sample size to achieve given power, you need to know several things, including what your outcome measure is, and how big the improvement in this outcome has to be to be considered clinically important. The latter is a clinical issue, not a statistical one, and as an expert in your field you will be in a better position to answer this than a statistician.” |
| **Cancer Research UK** | <http://www.cancerresearchuk.org/sites/default/files/egms_guidelines_-_prc_grant_applications_0.pdf> | “For each research question to be answered state the statistical analysis to be used, name the variables and describe the values. State the numbers of samples to be included in each analysis. Describe what can be achieved with this number of samples, including as appropriate the associated level of statistical power and be transparent about any potential limitations. Clarify other relevant details, either actual or expected, such as prevalence rates for biomarkers, numbers of events in clinical outcomes and length of follow-up for clinical outcomes. For research proposals using non-standard or non-well-known measures, a full copy of each measure must be included within an appendix to the application.” |
| **Wellcome Trust** | <http://www.wellcome.ac.uk/Funding/Innovations/Awards/Health-Innovation-Challenge-Fund/index.htm> | “All proposals submitted to the Health Innovation Challenge Fund must satisfy the following criteria.  Projects must have already demonstrated ‘proof-of-principle’ supported by experimental and, where feasible, in vivo data. Evidence from the applicant’s team must clearly illustrate the technical feasibility of the project and demonstrate the potential for development from its current state to a product approved for use in humans. Early stage research or discovery science is not fundable.  Proposals must include first testing in man during the concluding stages of the project and must have the potential to benefit patients within the following 3-5 years, having demonstrated efficacy and received the necessary regulatory approvals.” |
| **NIHR** | <http://www.hra.nhs.uk/resources/before-you-apply/clinical-study-design-considerations/> | Refers to HRA guidance |
| **FDA** | <http://www.fda.gov/RegulatoryInformation/Guidances/ucm126501.htm>  **Federal Regulation** [PART 314 -- APPLICATIONS FOR FDA APPROVAL TO MARKET A NEW DRUG](https://www.accessdata.fda.gov/scripts/cdrh/cfdocs/cfCFR/CFRSearch.cfm?CFRPart=314) Subpart D--FDA Action on Applications and Abbreviated Applications, Sec. 314.126 Adequate and well-controlled studies. | “The study uses a design that permits a valid comparison with a control to provide a quantitative assessment of drug effect. The protocol for the study and report of results should describe the study design precisely; for example, duration of treatment periods, whether treatments are parallel, sequential, or crossover, and whether the sample size is predetermined or based upon some interim analysis.” |
| **Health Canada** | <http://www.hc-sc.gc.ca/dhp-mps/prodpharma/applic-demande/guide-ld/ich/efficac/e6-eng.php> | “The number of subjects planned to be enrolled. In multicentre trials, the numbers of enrolled subjects projected for each trial site should be specified. Reason for choice of sample size, including reflections on (or calculations of) the power of the trial and clinical justification.”  Refers to: ICH HARMONISED TRIPARTITE GUIDELINE STRUCTURE AND CONTENT OF CLINICAL STUDY REPORTS  “Using the usual method for determining the appropriate sample size, the following items should be specified: a primary variable, the test statistic, the null hypothesis, the alternative ('working') hypothesis at the chosen dose(s) (embodying consideration of the treatment difference to be detected or rejected at the dose and in the subject population selected), the probability of erroneously rejecting the null hypothesis (the type I error), and the probability of erroneously failing to reject the null hypothesis (the type II error), as well as the approach to dealing with treatment withdrawals and protocol violations”  “The size of a trial is influenced by the disease to be investigated, the objective of the study and the study endpoints. Statistical assessments of sample size should be based on the expected magnitude of the treatment effect, the variability of the data, the specified (small) probability of error (see ICH E9) and the desire for information or subsets of the population or secondary endpoints |
| **European Commission**  **H2020** | <http://ec.europa.eu/health/human-use/clinical-trials/directive/index_en.htm>  <http://ec.europa.eu/health/files/eudralex/vol-1/reg_2014_536/reg_2014_536_en.pdf> | “a description of the statistical methods to be employed, including, if relevant:  — timing of any planned interim analysis and the number of subjects planned to be enrolled;  — reasons for choice of sample size”  “The members of the International Conference on Harmonisation of Technical Requirements for Registration of Pharmaceuticals for Human Use (ICH) have agreed on a detailed set of guidelines on good clinical practice which is an internationally accepted standard for designing, conducting, recording and reporting clinical trials, consistent with principles that have their origin in the World Medical Association's Declaration of Helsinki. When designing, conducting, recording and reporting clinical trials, detailed questions may arise as to the appropriate quality standard. In such a case, the ICH guidelines on good clinical practice should be taken appropriately into account for the application of the rules set out in this Regulation, provided that there is no other specific guidance issued by the Commission and that those guidelines are compatible with this Regulation” |
| **NIHR Statistics Group** |  | No specific relevant guidance |
| **CIHR** |  | No specific relevant guidance |
| **PCORI** | <http://www.pcori.org/research-results/research-methodology/pcori-methodology-standards>  <http://www.pcori.org/funding-opportunities/how-apply/have-question/chronic-low-back-pain-pfa-applicant-faqs> | “**RC-3: Power and sample size estimates must use appropriate methods to account for the dependence of observations within clusters and the degrees of freedom available at the cluster level.** The methods used to reflect dependence should be clearly described. Sources should be provided for the methods and for the data used to estimate the degree of dependence. Sensitivity analyses incorporating different degrees of dependence must be reported. For simpler designs, the dependence in the data can be reflected in the intraclass correlation. Dependence can also be reflected in variance components. Other factors that affect the power calculation and should be described include the design of the study, the magnitude of the hypothesized intervention effect, the prespecified primary analysis, and the desired Type I error rate. “  “PCORI does not require a minimum project sample size, as this will be up to the investigative team and will vary depending on the specific project. However, as noted in the PFA, "The studies must be relatively large, in part to be able to demonstrate differences in comparative effectiveness in the study arms as randomized, but also to allow adequate power to detect the potential differences in treatment responses in patient subgroups." We note that estimating sample size sufficient to provide adequate power for subgroup analysis can be challenging and typically requires expert consultation. Other potential challenges to achieving target sample size (e.g., unintended crossover) should also be factored into sample size considerations as appropriate.” |
| **NIH** | <http://www.nimh.nih.gov/research-priorities/policies/enhancing-the-reliability-of-nimh-supported-research-through-rigorous-study-design-and-reporting.shtml> | Generic  “Justification of sample size, including power calculations, number of subjects per condition, and a clear definition of “a subject” (e.g., in electrophysiological studies, is “one subject” the recording from one cell or the recording from one animal?) |
| **NHMRC** | <https://www.australianclinicaltrials.gov.au/researchers/research-principles-and-guidelines> | Generic  “The number of subjects planned to be enrolled. In multicentre trials, the numbers of enrolled subjects projected for each trial site should be specified. Reason for choice of sample size, including reflections on (or calculations of) the power of the trial and clinical justification.” |
| **AHRQ** |  | No specific relevant guidance |
